# Two Rare Cases of Feline Toxic Epidermal Necrolysis: A Novel Therapeutic Approach With Medical-Grade Honey

**DOI:** 10.1155/2024/2415811

**Published:** 2024-10-04

**Authors:** Alexandra Peteoacă, Niels A. J. Cremers, Linsey J. F. Peters

**Affiliations:** ^1^Faculty of Veterinary Medicine, University of Agronomic Sciences and Veterinary Medicine of Bucharest, 105 Splaiul Independentei, Bucharest, Romania; ^2^Department of Gynecology and Obstetrics, Maastricht University Medical Centre, Maastricht, Netherlands; ^3^R&D Department, Triticum Exploitatie BV, Sleperweg 44 6222 NK, Maastricht, Netherlands

## Abstract

Toxic epidermal necrolysis (TEN) is an acute and life-threatening dermatological condition that is drug-induced and characterized by extensive epidermal detachment. These lesions should be protected from infection using a product that has a low risk of reactivity. Medical-grade honey (MGH) exerts antimicrobial and wound-healing effects while posing a low risk of exacerbating TEN. In this case report, we are the first to describe the use of MGH for wound management in two feline TEN patients. Case 1 involved a 1-year-old female British shorthair cat, while Case 2 featured a 1-year-old female mixed-breed cat. Both patients presented to the hospital with various symptoms, including fever, tongue lesions, and lesions in the abdominal area following ovariohysterectomy surgery. TEN was confirmed via histopathological examination. The suspected cause of TEN in both cases was the iodine present in the surgical scrub. Tongue lesions were addressed with a liquid diet, and all xenobiotics were immediately withdrawn as a precaution. MGH products supplemented with vitamins were applied to the abdominal wounds following a wound lavage with Ringer's lactate. Daily dressing changes were performed without discomfort, pain, or any adverse reactions. In both cases, debridement became evident after just 1 day, and the patients fully recovered after 12 days of MGH therapy. This case report demonstrates for the first time the successful use of supplemented MGH for wound management in veterinary patients with TEN. The patients did not have any adverse reactions to the treatment, while MGH dressings provided antimicrobial protection and wound-healing effects. Furthermore, these cases highlight the importance of rapid diagnosis and immediate drug withdrawal to increase the survival rate. Overall, supplemented MGH is a safe and effective method to treat TEN-related lesions in feline patients.

## 1. Introduction

Toxic epidermal necrolysis (TEN) is a rare, severe, and potentially fatal dermatological condition occurring in both humans and animals [1]. This drug-induced pathology is characterized by rapid, widespread keratinocyte death and extensive epidermal detachment, although the pathogenesis of TEN in animals remains largely unclear [1, 2]. TEN is categorized under immune-mediated diseases and shares similarities with Stevens–Johnson syndrome (SJS). Clinical features of affected cats often include fever, lethargy, anorexia, and the development of characteristic mucosal and skin lesions [2]. Prompt and accurate diagnosis in such cases is critical, as delayed intervention may lead to systemic complications and increased mortality rates. Identification and immediate withdrawal of the causative drug are the first therapeutic steps [3]. This is followed by pain alleviation, fluid therapy, antibiotics, and symptomatic treatment for mucosal and skin lesions [3]. An ideal wound care product should have no reactivity and exert antimicrobial, anti-inflammatory, and prohealing activities.

The use of honey for the treatment of various medical conditions, especially wounds, has gained interest over the past two decades. Honey intended for medical purposes should, however, adhere to rigorous production and processing criteria [4]. This type is labeled as medical-grade honey (MGH) and possesses several wound-healing and antimicrobial properties [5, 6]. Various commercially available MGH products are available on the market for both human and veterinary use, including supplemented MGH. Several studies have demonstrated the synergistic effects between MGH and supplemented compounds, such as PEG 4000 or vitamins C and E [5, 7–10]. Supplementation of these compounds led to significantly stronger antimicrobial, antibiofilm, and prohealing effects compared to nonsupplemented MGH.

The use of supplemented MGH has also been shown in cats. This included the intrasocket application of supplemented MGH gel after tooth extractions in cats [11]. In this prospective randomized controlled trial, a split-mouth design was followed in which each cat served as its control. The single intrasocket application of MGH enhanced wound healing, reduced redness, and improved surgical flap viability [11]. Supplemented MGH previously led to complete skin regeneration after skin loss on the entire circumference of a leg in a cat [12]. Also, in a case series of 10 cats with 15 contaminated nonsurgical wounds, supplemented MGH had a positive impact on wound healing without any adverse effects of the MGH therapy [13]. However, to date, no reports have been made for the use of MGH to treat feline TEN. The aim of this clinical case report is to describe the clinical features and management of TEN in feline patients. In these two cases, a novel treatment method using supplemented MGH was applied to the wounds.

## 2. Case 1

**Figure 1:**
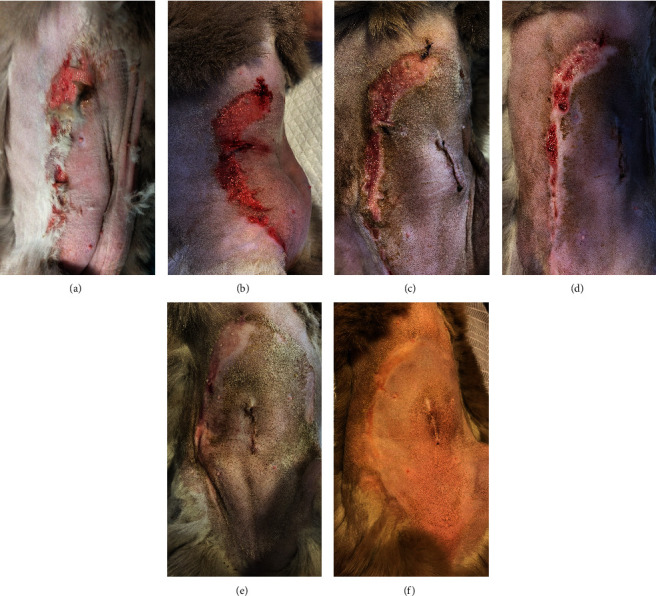
Wound healing progression in Case 1. (a) The abdominal wounds at the start of the MGH therapy (Day 0). (b) One day after the start of the treatment, the lesions were fully debrided. (c) On Day 4 of the MGH therapy, epithelialization of the wounds became evident. (d) Re-epithelialization continued, and the wound area further reduced (Day 7). (e) On Day 10, the lesions almost fully closed. (f) The skin showed minimal scarring 22 days after the start of the MGH therapy.

A 1-year-old female British shorthair cat presented to the clinic with a fever, superficial lesions in the tongue, and epidermal lesions in the abdominal area (Figure 1(a)). The patient underwent a routine ovariohysterectomy procedure 3 days before this. The surgical site was closed using a simple continuous pattern, first suturing the peritoneum and muscle layer, followed by an intradermic suture. Monofilament, absorbable suture material (polydioxanone) was used for both sutures, and the patient received amoxicillin/clavulanic acid 12.5 mg/kg subcutaneously. The patient's physiological values were within normal limits upon discharge.

Three days after the surgery, the patient was brought back to the emergency room with a temperature of 39.7°C. Ultrasonographic examination did not reveal any abnormalities. However, superficial erosions were observed on the margins of the tongue. PCR testing was conducted for calicivirus and various other infectious and parasitic diseases, all of which came back negative. Blood work showed slight abnormalities, including a mild leukocytosis, but was otherwise normal. Upon closer examination of the abdomen, well-demarcated epidermal lesions were observed, distinct from the healthy skin. In areas where the epidermal layer was destroyed, the wound exhibited the typical characteristics of redness and slight hemorrhaging. However, a larger area was affected superficially, that is, the first layers of the epidermis, and desquamation occurred in the following days. TEN was suspected, and a biopsy sample confirmed the diagnosis.

**Figure 2:**
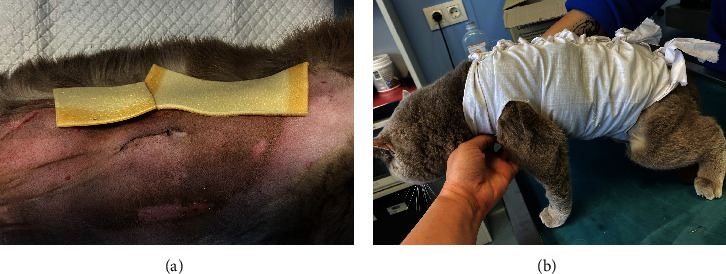
Bandaging of the abdominal wounds with the MGH-based foam dressing (a) and Alfort shirt (b) in Case 1.

To manage the tongue lesions, the patient was provided with a liquid diet for the first week. In consideration of the association between TEN and drug administration, the antibiotics were discontinued and no NSAIDs were prescribed for the pain. The surgical wound showed appropriate healing with no signs of dehiscence. The underlying tissues (subcutaneous and muscle) appeared more indurated than normal. Local wound management involved clipping the area to facilitate observation and lavaging with Ringer's lactate solution. An MGH-based foam dressing (L-Mesitran Foam, Triticum Exploitatie BV, the Netherlands) was applied directly to the wound surface using nonwoven gauze, cast padding, and self-adhesive wrap to secure it in place (covered by an Alfort shirt) (Figures 2(a) and 2(b)). The bandage was changed daily, alternating between the MGH-based foam dressing, an MGH-based wound contact layer (L-Mesitran Net, Triticum Exploitatie BV, the Netherlands), and MGH wound gel (L-Mesitran Soft, Triticum Exploitatie BV, the Netherlands). Since the lesions were limited to the epidermal layer and not full thickness, healing primarily occurred through epithelialization, considering the intact basement membrane. Pain management (buprenorphine) was required only during the two to three initial bandage changes, causing minimal discomfort to the patient. Pain was not experienced when removing bandages or cleaning the wound.

The day after the start of the MGH therapy, the lesions were fully debrided and a healthy wound bed was present (Figure 1(b)). On Day 4, re-epithelialization became evident as the wound area reduced, and this further progressed over the next days (Figures 1(c), 1(d), and 1(e)). By Day 12, the patient had completely healed, with the epidermal lesions resolved and normal skin regeneration achieved. The skin showed minimal scarring 22 days after the start of the MGH therapy (Figure 1(f)).

## 3. Case 2

**Figure 3:**
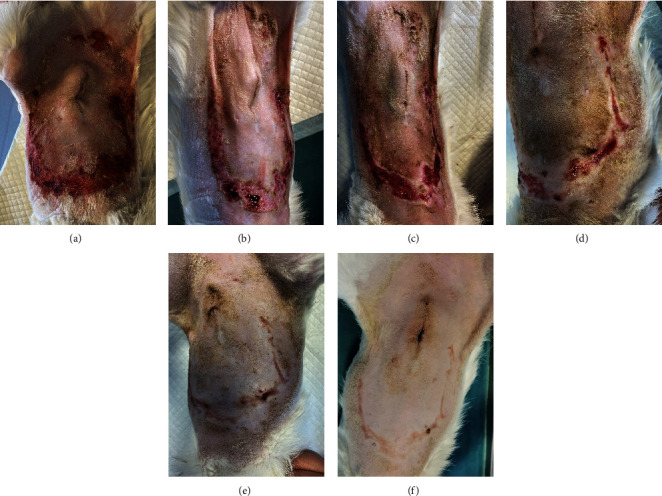
Wound healing progression in Case 2. (a) The abdominal wounds at the start of the MGH therapy (Day 0). (b) One day after the start of the treatment, the abdominal wounds were debrided and the smaller wound already started closing. (c) On Day 4, the debridement advanced and a healthy wound bed showed. The smaller wounds were fully healed. (d) Eight days after the start of the MGH therapy, the wound size decreased markedly. (e) On Day 11, the wounds were almost all fully healed. (f) Twenty-nine days after the start of the MGH treatment, the skin showed minimal scarring.

A 1-year-old female mixed-breed cat presented to the clinic with epidermal lesions in the abdominal area following ovariohysterectomy surgery (Figure 3(a)). PCR testing for calicivirus was performed and yielded negative results. The blood work revealed slight abnormalities, including mild leukocytosis and thrombocytopenia, although other parameters were within normal ranges. During the postoperative examination, well-demarcated epidermal lesions were observed on the patient's ventral abdomen. These lesions appeared as an oval-shaped, erosive area that was limited to the trimmed area from the previous surgery. The median line and the middle abdomen remained unaffected. The epidermal necrosis was suspected to be TEN, and a biopsy sample was collected for histopathological confirmation.

**Figure 4:**
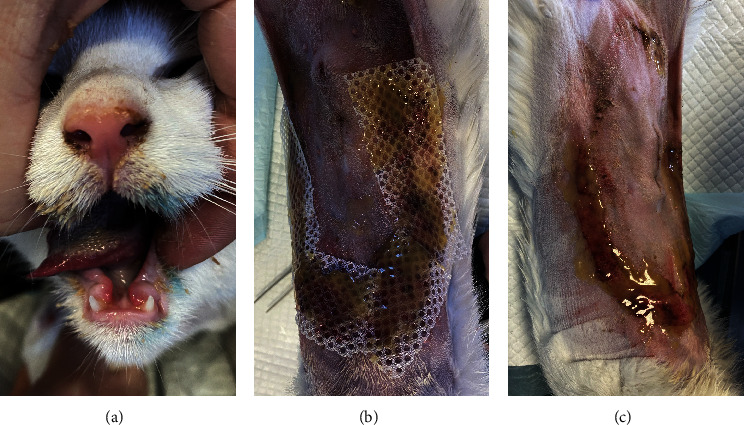
(a) The patient's tongue lesions. (b, c) Bandaging of the TEN wounds with the MGH-based wound gel in Case 2.

The patient presented to the clinic with a slightly increased temperature of 39.5°C, which normalized in less than 48 h. To address tongue lesions, the patient was placed on a liquid diet for the first week (Figure 4(a)). All medications were immediately discontinued (amoxicillin/clavulanic acid 12.5 mg/kg), considering the suspected association between TEN and drug administration. The surgical wound exhibited appropriate healing without signs of dehiscence. The subcutaneous and muscle tissues under the suture line appeared more indurated than normal. Local wound management involved careful clipping of the affected area to facilitate observation and Ringer's lactate solution lavage. The MGH foam dressing was directly applied to the wound surface using nonwoven gauze, cast padding, and self-adhesive wrap for secure placement. Daily bandage changes were performed, alternating between the MGH-based foam dressing, an MGH-based wound contact layer, and MGH wound gel (Figures 4(b) and 4(c)). The healing process primarily relied on epithelialization due to the lesions being confined to the epidermal layer, with some areas showing scar tissue formation where the lesions extended into the deeper dermis. Pain management was necessary during the initial bandage changes, causing minimal discomfort to the patient. No pain was reported during the removal of the previous bandages or wound cleaning. Slight excoriation was observed over the entire abdomen within the oval contour, without penetrating the vascularized layers of the skin.

On the first day following the start of the MGH therapy, debridement became evident in all lesions and the smaller lesions already started closing (Figure 3(b)). The smaller wounds had fully healed after 4 days. The larger wounds showed advanced debridement and initial re-epithelialization (Figure 3(c)). Over the next few days, the wounds continued to reduce in size without any further complications (Figures 3(d) and 3(e)). By Day 12, the patient had achieved complete healing, with resolution of the epidermal lesions. The skin showed minimal scarring 29 days after the start of the MGH therapy (Figure 3(f)).

## 4. Discussion

TEN is a rare and potentially fatal dermatological condition observed in both humans and animals [1]. Swift diagnosis in such cases is critical to reduce the chance of systemic complications and mortality. The initial therapeutic intervention is the withdrawal of the causative drug, which is followed by treatment of the lesions [3]. The current article reports two cases of feline TEN in which MGH-based products were applied to manage skin lesions.

Although TEN cases have not been extensively reported, few animal cases can be found in literature. For example, TEN was first reported in 1979 in two dogs and a cat, after which TEN was also found in other species, such as cattle and monkeys [14–16]. While the mortality rate of TEN in humans is estimated to be around 25%–35%, the mortality rate in animals remains unknown. However, based on the relatively few documented cases, mortality in animals is very high [2]. In another case of feline TEN, the patient developed rapidly deteriorating lesions on the seventh day of cefadroxil [17]. The cat was treated with intravenous fluids, antibiotics, and pain medication. Nonetheless, the owners decided euthanasia for the cat after 4 days of hospitalization as its condition rapidly worsened. The current two cases had a much more successful outcome compared to the other feline TEN report. This could be due to the fact that all medications, including antibiotics, were discontinued in the current cases, in contrast to cefadroxil-induced case. Although cefadroxil, an antibiotic, was discontinued, treatment with another antibiotic (marbofloxacin) was continued. Since antibiotics are responsible for over 25% of the total SJS and TEN cases worldwide, this could be a reason for the different outcomes [18].

Cases describing cats and dogs suffering from TEN consistently show the importance of prompt diagnosis and identification of the causative drug [17, 19–22]. Clinical signs of TEN are described as a sudden onset of disease with severe systemic signs like anorexia, lethargy, depression, and widespread skin and mucosal lesions. Epidermal detachment, which leads to extensive ulceration, is a characteristic feature of TEN. In cats and dogs, the main clinical signs are erosions and ulcers with a mostly truncal distribution. In most cases, the footpads are also affected, which was not the case in our patients. Mucosal ulcerations mainly involve the oral cavity but may also impact the cornea [2]. Notably, both of our patients showed truncal and tongue lesions, which correspond to the generally described signs. The symptoms, especially the lesions in the mouth, also align with the symptoms of calicivirus. However, tests were negative for any infectious diseases. This left TEN to be suspected as the cause, which was confirmed with a histopathological examination in both cases.

Identification of a candidate drug remains challenging because provocation testing is contraindicated and because there is no specific laboratory test to identify the involved drug [2]. Also in the current two cases, the causative drug remained elusive. For both patients, a potential association with the iodine in the scrub solution used during surgical preparation was considered, because of the shape and location of the lesions. Both cats developed a circular lesion on the ventral abdomen close to the border where the hair was clipped for surgery. Some cases of TEN/SJS in humans were caused by iodine present in contrast fluid [23, 24]. The iodine in the current cases was, however, applied topically which has not been reported before as a cause of TEN to our knowledge. Although the iodine scrub was considered the most likely cause, TEN could have also been caused by the administration of antibiotics. However, pinpointing the exact drug responsible was impossible. Histopathological examination confirmed the characteristic features of TEN. Based on the rapid diagnosis, all medications were discontinued. This could be done without major risk of complications due to the general state of the animal and it being a routine procedure. In humans, the identification of the causative drug is based on the algorithm for assessment of drug causality (ALDEN) score. It has been suggested to use an adapted version of the ALDEN score for animal TEN [2]. Some reported animal cases made use of this. The aforementioned feline TEN case was the only case using ALDEN in a cat [17]. Other cases demonstrated the use of the ALDEN scoring system in dogs. Notably, for only 2 out of 4 canine cases, the ALDEN score revealed the probable cause of TEN [19, 20]. This highlights the need for more frequent use of the scoring system in animals, thereby optimizing its application. In any TEN case, all xenobiotics need to be withdrawn immediately unless acute withdrawal poses a life-threatening risk [20].

One important aspect of wound care, and health care in general, is the costs that are related to the therapy. In the two presented TEN cases, the total costs for each patient summed up to around €70. The MGH-based products used for wound care averaged to around €35–€40, while the additional bandage materials costed around €30–€35. For the current scenario, the use of MGH resulted in average costs for treatment. In more severe wounds requiring longer treatment periods, the price of MGH, especially the wound gel variant, is lower compared to other products used for moist healing. While antimicrobial ointments like silver sulfadiazine are cheaper, they cannot be directly compared due to their different characteristics. Furthermore, it can be argued that the healing time should be considered in relation to the price. The longer the healing time, the higher the price. While cheaper products such as paraffin-impregnated nets, alginates, or other ointments exist, they may not be suitable for all healing phases and may not yield the same positive results. Therefore, despite their lower cost, they might not provide the same value in terms of overall healing efficacy compared to MGH. Some systematic reviews have also underlined the cost-effectiveness of MGH for wound care [25, 26]. However, to validate these observations, one should perform an overall cost-effectiveness analysis of MGH in future studies.

To our knowledge, in these cases, the skin lesions were treated with MGH for the first time in animals suffering from TEN. Only one other case has reported the use of MGH in a human TEN patient [27]. The skin lesions require a treatment that prevents infection while stimulating re-epithelialization. Besides this, the product of choice should not induce further reactions in the patient. Therefore, antibiotic ointments, povidone-iodine, or silver sulfadiazine should be avoided in TEN patients. MGH is an ideal wound care product for treating TEN lesions since it has a wide-spectrum antimicrobial effect while promoting wound healing in multiple manners. Furthermore, no honey-induced TEN case has been reported to date, and allergies to honey are uncommon and estimated to be <0.001% [28]. The honey used in medical wound care products also is of medical grade, thereby minimizing the risk of any adverse reactions [4]. MGH supplemented with vitamins has been used safely and effectively to treat wounds of different etiologies in cats. Previously, in a series involving 10 cats with a total of 15 contaminated nonsurgical wounds, the use of a supplemented MGH wound care product positively influenced the healing of wounds [13]. Furthermore, MGH supplemented with vitamins C and E resulted in the complete regeneration of skin following the loss of skin around the entire circumference of a cat's leg [12]. In none of these, or any other cases involving humans or other animal species, adverse effects of the supplemented MGH treatment were observed.

## 5. Conclusion

Both feline patients developed TEN following a routine ovariohysterectomy procedure. The patients responded well to the implemented treatment plan, which involved discontinuing medications, providing specialized dietetic food, and employing local wound management techniques. With appropriate wound care and time, complete healing was achieved, demonstrating the successful resolution of TEN in these cases. This highlights the importance of early recognition and prompt management of TEN in veterinary patients while acknowledging the need for further research to identify the specific etiological factors underlying this condition.

## Data Availability

The underlying data used to support the findings of this study are available from the corresponding author upon request.

## References

[1] Harris V., Jackson C., Cooper A. (2016). Review of toxic epidermal necrolysis. *International Journal of Molecular Sciences*.

[2] Yager J. A. (2014). Erythema multiforme, Stevens-Johnson syndrome and toxic epidermal necrolysis: a comparative review. *Veterinary Dermatology*.

[3] Schwartz R. A., McDonough P. H., Lee B. W. (2013). Toxic epidermal necrolysis. *Journal of the American Academy of Dermatology*.

[4] Hermanns R., Mateescu C., Thrasyvoulou A., Tananaki C., Wagener F. A., Cremers N. A. (2020). Defining the standards for medical grade honey. *Journal of Apicultural Research*.

[5] Cremers N., Belas A., Santos Costa S., Couto I., de Rooster H., Pomba C. (2020). In vitro antimicrobial efficacy of two medical grade honey formulations against common high-risk meticillin-resistant staphylococci and Pseudomonas spp. pathogens. *Veterinary Dermatology*.

[6] Maddocks S. E., Jenkins R. E. (2013). Honey: a sweet solution to the growing problem of antimicrobial resistance?. *Future Microbiology*.

[7] Majtan J., Sojka M., Palenikova H., Bucekova M., Majtan V. (2020). Vitamin C enhances the antibacterial activity of honey against planktonic and biofilm-embedded bacteria. *Molecules*.

[8] Pleeging C. C. F., Coenye T., Mossialos D. (2020). Synergistic antimicrobial activity of supplemented medical-grade honey against *Pseudomonas aeruginosa* biofilm formation and eradication. *Antibiotics*.

[9] Subrahmanyam M. (1998). A prospective randomised clinical and histological study of superficial burn wound healing with honey and silver sulfadiazine. *Burns*.

[10] de Groot T., Janssen T., Faro D., Cremers N. A. J., Chowdhary A., Meis J. F. (2021). Antifungal activity of a medical-grade honey formulation against *Candida auris*. *Journal of Fungi*.

[11] Pleeging C. C., de Rooster H., Van Wijk B., Wagener F. A., Cremers N. A. (2022). Intra-socket application of medical-grade honey after tooth extraction attenuates inflammation and promotes healing in cats. *Journal of Feline Medicine and Surgery*.

[12] Lukanc B., Potokar T., Erjavec V. (2020). Complete skin regeneration with medical honey after skin loss on the entire circumference of a leg in a cat. *Journal of Tissue Viability*.

[13] Lukanc B., Potokar T., Erjavec V. (2018). Observational study of the effect of L-Mesitran medical honey on wound healing in cats. *Veterinarski Arhiv*.

[14] Scott D. W., Halliwell R. E. W., Goldschmidt M. H., DiBartola S. (1979). Toxic epidermal necrolysis in two dogs and a cat. *Journal of the American Animal Hospital Association*.

[15] Garman R. H., Reed C., Blick D. W. (1979). Toxic epidermal necrolysis in a monkey (*Macaca fascicularis*). *Veterinary Pathology*.

[16] Yeruham I., Perl S., Elad D. (1999). Nine cases of idiopathic toxic epidermal necrolysis in cattle in Israel. *Journal of Veterinary Medicine, Series B*.

[17] Sartori R., Colombo S. (2016). Stevens-Johnson syndrome/toxic epidermal necrolysis caused by cefadroxil in a cat. *JFMS Open Reports*.

[18] Lee E. Y., Knox C., Phillips E. J. (2023). Worldwide prevalence of antibiotic-associated Stevens-Johnson syndrome and toxic epidermal necrolysis: a systematic review and meta-analysis. *JAMA Dermatology*.

[19] Banovic F., Olivry T., Bazzle L. (2015). Clinical and microscopic characteristics of canine toxic epidermal necrolysis. *Veterinary Pathology*.

[20] Lecru L. A., Combarros D., Castilla-Castano E., Delverdier M., Cadiergues M. C., Pressanti C. (2021). Case report: positive outcome of a suspected drug-associated (immune mediated) reaction in a 4-year-old male French bulldog. *Frontiers in Veterinary Science*.

[21] Nishiyama T., Iyori K., Iwasaki T., Nishifuji K. (2015). Detection of apoptotic epidermal cells in a dog with toxic epidermal necrolysis. *Japanese Journal of Veterinary Dermatology*.

[22] Nii A., Tashiro T., Sato Y. (2007). Erythroderma and epidermal necrosis induced by a type of proton pump inhibitor in beagle dogs. *Journal of Toxicologic Pathology*.

[23] Carrera D., Ulloa J. G. (2022). Iodinated contrast-induced Stevens-Johnson syndrome: a report of a rare complication for a common imaging agent. *Journal of Vascular Surgery Cases and Innovative Techniques*.

[24] Tasker F., Fleming H., McNeill G., Creamer D., Walsh S. (2019). Contrast media and cutaneous reactions. Part 2: delayed hypersensitivity reactions to iodinated contrast media. *Clinical and Experimental Dermatology*.

[25] Oryan A., Alemzadeh E., Moshiri A. (2016). Biological properties and therapeutic activities of honey in wound healing: a narrative review and meta-analysis. *Journal of Tissue Viability*.

[26] Yilmaz A. C., Aygin D. (2020). Honey dressing in wound treatment: a systematic review. *Complementary Therapies in Medicine*.

[27] Henry N., Jeffery S., Radotra I. (2019). Properties and use of a honey dressing and gel in wound management. *The British Journal of Nursing*.

[28] Aguiar R., Duarte F. C., Mendes A., Bartolome B., Barbosa M. P. (2017). Anaphylaxis caused by honey: a case report. *Asia Pacific Allergy*.

